# A Report on the Hygiene of the United States Army

**Published:** 1875-09

**Authors:** 


### Bibliographical.

A Report on the Hygiene of the United States Army, with descriptions of the military ports, is one of those documents popularly known as circulars, from the official orders prefacing them, that emanate from the Surgeon General's office. This is promulgated by circular 8, 1875.

About one hundred and eighty garrisoned places are carefully described, and from a study of them one can obtain a very fair notion of the medical geography of the country. Some of these reports discuss subjects which are of importance to civilians as well as to military men: as, for example, the Rocky Mountain region as a resort for consumptives; the effect of over-crowding and want of ventilation; food in its relation to work. And as they are made by intelligent men, who have no local prejudices to influence them, they are valuable contributions to sanitary science.

The preliminary Report on Hygiene is a brief but clear-treated paper on some important topics, drawn up by Assistant Surgeon Billings. We have only room for his rational remarks on hospitalism: "As regards deleterious gases this [free ventilation] will do very well; but the real danger of hospitalism arises from solid particles, probably living, and the results of attempting to dilute these have been and will continue to be * * [that] the probability of infection for one exposure may be diminished, but when the small particle does happen to be present, its effect will be the same as though no dilution had been attempted. * * it seems probable that they increase in violence by successive transmissions * * and I do not believe that any system of hospital construction or ventilation will prevent hospitalism which does not allow of a much more minute classification of cases than is now practicable, and in which ample provision is not made for the isolation of cases when needed."

The volume is a credit to the profession, and especially to the Army Medical Department; and is one of those occasional publications that add to the respect felt for this branch of the public service.

